# A Case Report on Schistosoma and Gastric Cancer: Association or Causation?

**DOI:** 10.7759/cureus.85626

**Published:** 2025-06-09

**Authors:** Hamzah El Baba, Aamer Hassan Hamed, Syed Rezvi, Ahmed Suliman, Ahmed Z Al-Bahrani

**Affiliations:** 1 Department of General Surgery, Hamad Medical Corporation, Doha, QAT; 2 Department of Upper GI (Gastrointestinal) Surgery, Hamad Medical Corporation, Doha, QAT; 3 Department of Pathology, Hamad Medical Corporation, Doha, QAT

**Keywords:** diffuse type, gastric cancer, schistosoma, schistosoma mansoni, stomach neoplasms

## Abstract

The association of gastric cancer with parasitic infestation is rather uncommon. We report a case of gastric cancer accompanied by *Schistosoma (S.) mansoni*, incidentally found in histopathological examination. While this coexistence may be incidental, the rarity of such a finding prompts consideration of a possible pathophysiological link. A 39-year-old Sudanese man presented with epigastric pain, significant weight loss of 20 kg over six months, and dyspepsia, without nausea or vomiting. His medical history included diabetes mellitus and previous tobacco use. Laboratory investigations revealed anemia, hypoalbuminemia, and elevated tumor markers, including carcinoembryonic antigen (CEA) and cancer antigen 125 (CA 125). Endoscopic evaluation identified a nodular, friable mass with ulceration on the lesser curvature of the stomach, and biopsy confirmed signet-ring cell adenocarcinoma with *Schistosoma* infestation. Immunohistochemistry revealed HER2 negativity, intact mismatch repair (MMR), and PD-L1 positivity. Computed tomography (CT) imaging demonstrated advanced gastric cancer (cT4cN3M1). Management included palliative chemotherapy, genetic counseling, potential pyloric stenting, and treatment of schistosomiasis. This case illustrates the exceedingly rare histological coexistence of *S. mansoni* and diffuse-type gastric cancer, underscoring the complex and poorly understood relationship between infectious agents and gastric malignancy. Further research is warranted to elucidate the potential role of *S. mansoni* in gastric cancer pathogenesis.

## Introduction

Gastric cancer (GC), ranked as the fifth most prevalent malignancy globally, is associated with the third highest fatality rates across genders [1]. It is categorized into intestinal, diffuse, and mixed types. Moreover, the Japanese Gastric Cancer Association's proposed classification system delineates five prevalent types of gastric carcinoma: papillary adenocarcinoma, tubular adenocarcinoma, poorly differentiated adenocarcinoma, signet ring cell carcinoma, and mucinous carcinoma [2]. Risk factors for gastric cancer are multifaceted and can be categorized into three primary domains: environmental factors, genetic predisposition, and the role of infectious agents. Among these agents, Epstein-Barr virus (EBV) and *Helicobacter pylori (H. pylori)* hold notable prominence [3].

Schistosomiasis, formerly referred to as bilharzia, is a condition originating from parasitic infestation by flukes belonging to the *Schistosoma (S.) *genus [4]. The human population is primarily affected by five key schistosome species: *S. japonicum, S. haematobium, S. mekongi, S. intercalatum, *and* S. mansoni *[5]. The clinical manifestation of chronic schistosomiasis can vary depending on the specific *Schistosoma* species involved. Independent research studies have demonstrated that infections caused by distinct species of *Schistosoma* can give rise to various forms of cancer [6,7]. Urogenital schistosomiasis induced by *S. haematobium* stands as a recognized carcinogen, constituting the second most common cause of global bladder cancer cases [8]. A comprehensive correlational investigation conducted in China examined the linkage between mortality attributed to *S. japonicum* infection and mortality arising predominantly from colorectal, liver, esophageal, and gastric cancers [9]. Several case reports have documented the occurrence of liver cancer, colorectal cancer, giant follicular lymphoma, and various other types of cancers in connection with *S. mansoni *infestation [10]. However, it's important to note that no case has reported any connection between *S. mansoni* infection and gastric cancer.

To our knowledge, this is the first reported case of diffuse-type gastric adenocarcinoma (linitis plastica) associated with *Schistosoma mansoni* infestation.

This case report is presented in accordance with the updated consensus-based Surgical Case Report (SCARE) guidelines [11].

## Case presentation

A 39-year-old Sudanese man presented to the upper GI surgery outpatient clinic of our institution (Hamad General Hospital, the largest tertiary facility in Doha, Qatar) in August 2023, complaining of epigastric pain, left upper quadrant pain, and unexplained persistent recent-onset dyspepsia lasting more than six weeks. He reported no nausea or vomiting but mentioned a weight loss of approximately 20 kilograms over six months, along with episodes of hematemesis and melena. The patient adapted to smaller meals and experienced dark stools after initiating iron supplementation. He had a history of diabetes mellitus, which was managed with metformin, and a prior tonsillectomy. His social history included being an ex-smoker for six years and never consuming alcohol. Physical examination revealed vital signs within normal ranges: temperature of 36.7 °C, heart rate of 82 beats per minute (bpm), respiratory rate of 19 breaths per minute, blood pressure of 111/68 mmHg, and oxygen saturation of 100%. His weight was recorded as 64 kg with a calculated body mass index of 19.5 kg/m^2^. No pallor or jaundice was observed. Mild epigastric fullness was noted while the rest of the abdominal examination was unremarkable.

**Figure 1:**
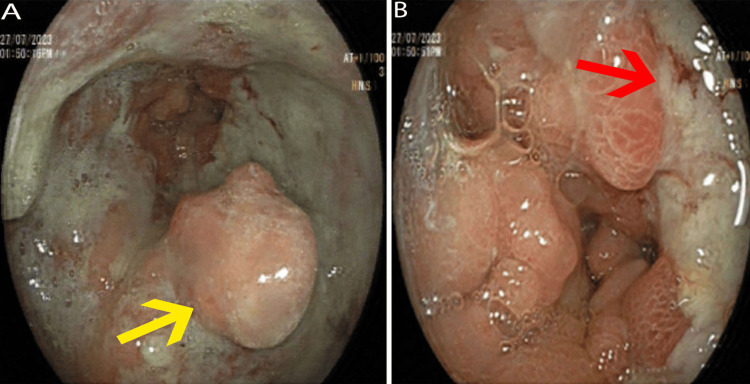
Esophagogastroduodenoscopy (A, B) A) A nodular, friable mass is seen on the lesser curvature of the stomach (yellow arrow); B) Circumferential involvement of the distal gastric body and antrum, with mucosal ulceration, friability, and luminal narrowing (red arrow) and scope could not be passed into the duodenum

He had an ultrasound abdomen, which revealed a thickened stomach wall, particularly in the epigastric region, with multiple hypoechoic structures. The largest of these structures measured 20 x 15 mm. These ultrasound findings suggested a thick-walled stomach with potential lymph nodes within the epigastric region. Laboratory results showed that the patient's hemoglobin (reference: 13.5-17.5 g/dL) was measured at 8.8 g/dL. Additionally, his albumin level was 28 g/L, carcinoembryonic antigen (CEA) (reference: <5 ng/mL for non-smokers) of 202 ng/mL, CA 125 (reference: <35 U/mL) of 64 U/mL, and cancer antigen 19-9 (reference: <37 U/mL) of 6.3 U/mL. Electrolyte, renal function, and liver function tests were within normal ranges. An esophagogastroduodenoscopy (OGD) revealed a nodular friable mass involving the lesser curve of the stomach, where a biopsy was taken. The mass extended from just below the gastroesophageal (GE) junction, with the distal body and antrum exhibiting narrowing, ulceration, and friability that prevented scope passage into the duodenum (Figures 1A, 1B).

**Figure 2:**
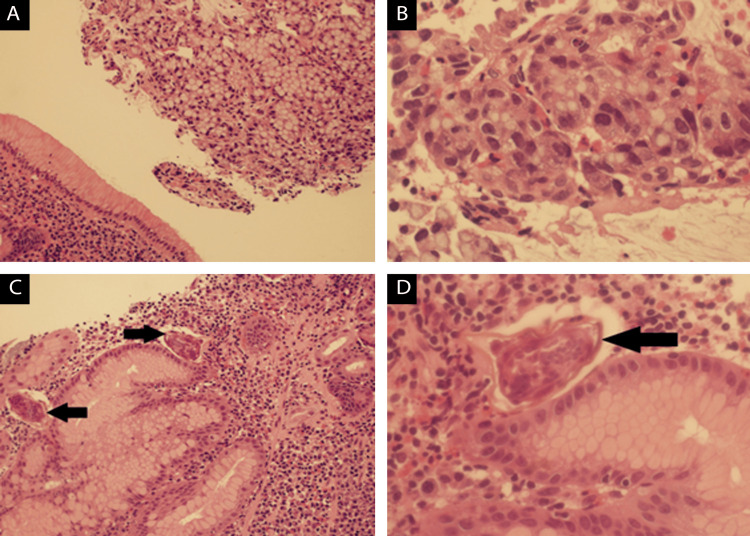
Histopathological examination indicating gastric signet-ring cell adenocarcinoma with Schistosoma mansoni infestation A) Low-power view (2× objective) of gastric mucosal biopsy showing normal gastric foveolar epithelium (bottom left) with diffuse signet-ring cell carcinoma morphology (top right); B) High-power view of diffuse-type gastric carcinoma with admixed eosinophils (an unusual feature); C) Adjacent foveolar epithelium containing *Schistosoma* organisms (indicated by black arrows); D) *Schistosoma* with a lateral spine, characteristic of *Schistosoma* *mansoni* (indicated by black arrows).

Histological analysis of a gastric mass biopsy confirmed signet-ring cell adenocarcinoma (Figures 2A, 2B) with a *Schistosoma mansoni* infestation (Figures 2C, 2D). Importantly, the HER-2 status was negative (score +1). Moreover, there was no loss of nuclear expression of mismatch repair proteins (MMR), indicating a low probability of microsatellite instability (MSI-H). Furthermore, the tumor exhibited a positive expression of programmed death-ligand 1 (PD-L1), with a combined positive score (CPS) greater than 1, suggesting potential eligibility for immunotherapy.

**Figure 3:**
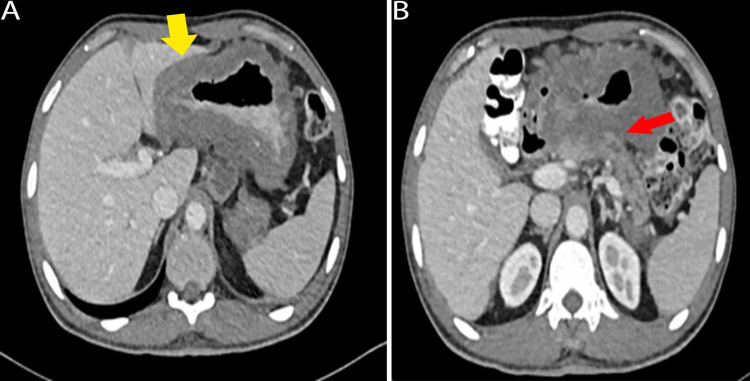
Axial CT of the chest, abdomen, and pelvis with oral and IV contrast (A, B) A) Diffuse thickening of the gastric wall, especially at the lesser curvature, is noted, appearing inseparable from the adjacent hepatic parenchyma (yellow arrow); B) The mass extends to infiltrate the adjacent pancreatic parenchyma (red arrow), with multiple perigastric lymph nodes and numerous omental and peritoneal nodules

A contrast-enhanced CT scan of the chest, abdomen, and pelvis (Figures 3A, 3B) was conducted to stage his gastric cancer and revealed diffuse thickening of the gastric wall, especially at the lesser curvature, with a maximum thickness of 28 mm. Multiple perigastric and coeliac lymph nodes were noted, with the largest measuring 22 x 26 mm and central necrosis. The mass extended beyond the gastric serosa, infiltrating adjacent pancreatic parenchyma mostly at the body, with distal pancreatic atrophy and slight ductal prominence. It was inseparable from the adjacent hepatic parenchyma and transverse colon, indicating a loss of fat planes. Omental, peritoneal, and mesenteric nodules were present, including the largest at the Douglas pouch (38 x 16 mm). Hypodense regions on the right hepatic lobe suggested peritoneal deposits. The pancreas showed direct infiltration (28 x 18 mm), with mild ductal prominence and parenchymal atrophy. Small para-aortic lymph nodes were also detected.

The patient was diagnosed with gastric adenocarcinoma diffuse type (Linitis Plastica), staged as cT4cN3M1, grade 2 with *Schistosoma *(Mansoni)infestation. In light of this diagnosis, the management plan, devised by the upper GI multidisciplinary team, encompasses a comprehensive approach. It includes genetic counseling, palliative chemotherapy +/- immunotherapy, and the potential implementation of pyloric stenting is also being considered as a measure to alleviate gastric outlet narrowing and optimize gastric function. Additionally, the infectious diseases team initiated treatment with praziquantel: 1,200 mg, oral, BID for one day, targeting the concurrent *Schistosoma* (Mansoni) infestation. This approach reflects a concerted effort to address all relevant aspects of the patient's condition.

The patient was closely followed by the oncology team and received nine cycles of FOLFOX chemotherapy (folinic acid, fluorouracil, and oxaliplatin) in combination with pembrolizumab. The regimen was initially well-tolerated, with no reported complications. The FOLFOX protocol included intravenous oxaliplatin (85 mg/m²), leucovorin (200 mg/m²), and fluorouracil (2,600 mg/m² as a 24-hour infusion). Despite treatment, the patient’s condition gradually deteriorated, and he died one year after diagnosis due to disease progression.

## Discussion

Stomach cancer shows marked geographic and gender-related variations in incidence, with rates approximately twice as high in men as in women and highest in Eastern Asia, Central and Eastern Europe, and South America [12]. Regional variations partly result from differences in dietary patterns, food storage, and the availability of fresh ingredients, as well as the prevalence of *Helicobacter pylori *infection [13]. Notably, chronic infection with *H. pylori* is the strongest identified risk factor for stomach cancer, with about 90% of new cases of non-cardia gastric cancer worldwide [14].

Gastric carcinoma is a malignancy of high aggressiveness with its heterogeneous nature [15]. That is why alternative prevention, considered as a proper diet, early diagnosis, follow-up, and proper treatments, leads to the reduction of recorded incidents [16]. Gastric cancer is rather rare and is not prevalent in the young population (under 45 years of age) [17]. Our patient is African in origin, only 39 years old, with a negative *H. pylori* infection, but a biopsy showed *Schistosoma mansoni* infestation associated with the gastric cancer.

As for clinical presentation, gastric cancer often presents with vague and non-specific symptoms, including epigastric pain, palpable mass, unexplained weight loss, anorexia, dysphagia, nausea, and vomiting. Such non-specificity frequently leads to delays in diagnosis [18]. This is the case with our patient who presented with dyspepsia and non-specific symptoms, and he presented late with more advanced disease.

As for investigations and staging, endoscopic ultrasound (EUS) and CT scan are widely used to evaluate tumor depth (T) and regional lymph node involvement (N). EUS, especially when combined with endoscopy, allows detailed visualization of tumor infiltration depth and can facilitate targeted biopsy sampling [19,20]. Furthermore, the CT of the thorax and abdominopelvic region is the principal modality to evaluate the M stage, with the sensitivity and specificity ranging from 14% to 59.1% and 93.3% to 99.8% [21]. In our patient, OGD revealed a narrowed stomach with a mass along the lesser curvature. A staging CT scan revealed advanced disease, including peritoneal deposits, local invasion into the colon, pancreas, and liver, as well as nodal metastases. Given the extent of disease evident on CT and histological confirmation, additional staging procedures, such as EUS, PET/CT, or staging laparoscopy, were not deemed necessary in this case. Notably, 18F-fludeoxyglucose (FDG) positron emission tomography (PET)/computed tomography (CT) shows lower sensitivity and specificity in detecting diffuse-type gastric cancers (63% and 60%, respectively) compared to non-signet-ring subtypes (75% and 75%) [22]. A review by Dondi et al. found that diffuse gastric cancer typically has lower FDG uptake compared to other histotypes [23].

Histologically, gastric adenocarcinoma can be divided into two histological groups: intestinal and diffuse. The incidence of the intestinal type of gastric carcinoma is decreasing. However, it remains the most common form of gastric carcinoma [24]. Gastric linitis plastica is a subtype of diffuse gastric carcinoma, making up 7-10% of gastric adenocarcinomas, characterized by thickened gastric walls and infiltrating signet ring cells [25]. These findings align with our case in which histopathological examination revealed diffuse-type gastric carcinoma with a predominance of carcinoma cells exhibiting a signet ring morphology. Interestingly, the examination also identified the presence of eosinophils within the tumor microenvironment, an uncommon feature, along with adjacent foveolar epithelium displaying Schistosomes with lateral spines, indicative of *Schistosoma mansoni* infestation.

In the context of the previously discussed histopathological findings, it is noteworthy that schistosome infestation may serve as a potential initiator or promoter of carcinogenesis. This propensity arises from their sustained presence within the host, particularly during the chronic phase of infection [26]. Currently, seven *Schistosoma *species have been identified as etiological agents of human infestation. Among these, *S. haematobium*, *S. mansoni*, and *S. japonicum* are the most prevalent and have been associated with various degrees of carcinogenicity, contributing to different forms of cancer [27].

In regions endemic to *S. haematobium*, the parasitic infection is directly linked to approximately 46-75% of all cases of bladder squamous cell carcinoma, and as a result, *S. haematobium *is classified as a Group 1 carcinogen by the International Agency for Research on Cancer (IARC) [7]. *Schistosoma japonicum* has been designated as a probable human carcinogen (Group 2B) by the International Agency for Research on Cancer due to its association with the development of liver cancer [28]. Furthermore, there is accumulating pathological evidence suggesting a potential link between *S. japonicum* infection and the onset of colorectal carcinogenesis and its prognosis [29]. In addition, two studies done in China found an association between *Schistosoma japonicum* and gastric cancer [30,31].

The available scientific evidence regarding the relationship between *S. mansoni *infection and cancer is currently inadequate [32]. This ambiguity in the data is reflected in the International Agency for Research on Cancer's classification of *S. mansoni* within Group 3, denoting a lack of sufficient evidence to conclusively establish its carcinogenic potential [33]. Nevertheless, observational case reports and descriptive studies conducted in endemic areas have explored the potential correlation between *S. mansoni *infestation and various types of cancer, including colorectal cancer [34], hepatocellular carcinoma [35], bladder carcinoma [36], follicular lymphomas [37], and prostate cancer [38]. Additionally, after an extensive search, one study reported a coincidence of *Schistosoma mansoni* with intestinal-type gastric adenocarcinoma [39]. To date, no reported cases have established an association between *Schistosoma mansoni* and diffuse-type gastric cancer. Hence, our case represents the first reported case of such an association.

Regarding management and prognosis, patients with linitis plastica of the stomach have a poor prognosis with a five-year survival of 3-10% in various studies [40]. The management of this case aligned with the recommendations of our Upper Gastrointestinal Multidisciplinary Team, which advised palliative chemotherapy. The patient was treated with FOLFOX chemotherapy in combination with pembrolizumab; FOLFOX has demonstrated a 43.8% overall response rate, with a median progression-free survival of 6.0 months and overall survival of 12.6 months, and is generally well-tolerated [41]. Additionally, the patient was followed by the oncology team throughout his treatment and received praziquantel for *Schistosoma* infection under the care of the infectious diseases team. Despite these interventions, his condition gradually deteriorated, and he passed away one year after diagnosis due to disease progression.

## Conclusions

We present the first case of diffuse-type gastric carcinoma coinciding with *Schistosoma mansoni* infection. This association underscores the potential interplay between infectious agents and cancer development and serves as a valuable addition to the limited body of literature. Overall, this case highlights the need for further exploration of the potential role of *Schistosoma mansoni* infections in carcinogenesis, especially in the context of gastric malignancies.
